# Maintenance of human haematopoietic stem and progenitor cells in vitro using a chemical cocktail

**DOI:** 10.1038/s41421-018-0059-5

**Published:** 2018-10-30

**Authors:** Mengmeng Jiang, Haide Chen, Shujing Lai, Renying Wang, Yunfei Qiu, Fang Ye, Lijiang Fei, Huiyu Sun, Yang Xu, Xinyi Jiang, Ziming Zhou, Tingyue Zhang, Yanwei Li, Jin Xie, Qun Fang, Robert Peter Gale, Xiaoping Han, He Huang, Guoji Guo

**Affiliations:** 1 Center for Stem Cell and Regenerative Medicine, Zhejiang University School of Medicine, Zhejiang, Hangzhou, 310058 China; 2 Stem Cell Institute, Zhejiang University, Zhejiang, Hangzhou, 310058 China; 3 Institute of Hematology, First Affiliated Hospital, Zhejiang University School of Medicine, Zhejiang, Hangzhou, 310003 China; 4 UT-ORNL Graduate School of Genome Science and Technology, The University of Tennessee, Knoxville, TN 37996 USA; 5 Core Facilities, Zhejiang University School of Medicine, Zhejiang, Hangzhou, 310058 China; 6 Institute of Mechatronic Control Engineering, Zhejiang University, Zhejiang, Hangzhou, 310027 China; 7 Department of Chemistry, Institute of Microanalytical Systems, Zhejiang University, Zhejiang, Hangzhou, 310058 China; 8 Department of Medicine, Haematology Research Centre, Division of Experimental Medicine, Imperial College London, London, UK; 9 Alliance for Atlas of Blood Cells, Hangzhou, China; 10 Zhejiang Provincial Key Lab for Tissue Engineering and Regenerative Medicine, Dr. Li Dak Sum & Yip Yio Chin Center for Stem Cell and Regenerative Medicine, Zhejiang, Hangzhou, 310058 China

## Abstract

Identification of effective culture conditions to maintain and possibly expand human HSPCs in vitro is an important goal. Recent advances highlight the efficacy of chemicals in maintaining and converting cell fates. We screened 186 chemicals and found that a combination of CHIR-99021, Forskolin and OAC1 (CFO) maintained human CD34-positive cells in vitro. Efficiency of the culture system was characterized using flow cytometry for CD34-positive cells, a colony-forming assay and xeno-transplants. We found that human CD34-positive cells treated with this combination had enhanced expression of human HSPC markers and increased haematopoietic re-populating ability in immune-deficient mice. Single-cell RNA-seq analyses showed that the in vitro cultured human CD34-positive cells were heterogeneous. We found that CFO supports maintenance of human CD34-positive cells by activating *HOXA9*, *GATA2* and AKT-cAMP signaling pathway. These data have implications in therapies requiring maintenance and/or expansion of human HSPCs.

## Introduction

Identification of effective culture conditions to maintain and possibly expand human HSPCs ex vivo is an important goal for hematological researches. Previous studies tried to optimize culture conditions with haematopoietic growth factors (HGFs) and exogenous gene expressions to maintain and expand human HSPCs in vitro. However, these attempts are mostly unsuccessful^1–3^. Low molecular weight chemicals can initiate cell re-programming in diverse systems^4^. Pluripotent stem cells can be obtained from mouse fibroblast, neural stem cells and small intestinal epithelial cells using low molecular weight chemicals^5,6^. We reported that mouse embryonic fibroblasts can be trans-differentiated into diverse somatic lineages following treatment with a combination of chemicals^7^. In addition, cardiomyocyte-like cells can be generated by treating human fibroblasts with several small molecular weight chemicals^8^. These chemicals can also expand adult stem cells including inducing proliferation of mature primary human hepatocytes and converting rat and mouse mature hepatocytes to proliferative, bi-potent cells in vitro^9,10^.

Similar data were reported in the context of human HSPCs. Boitano et al. reported that SR1, an aryl-hydrocarbon-receptor antagonist, promotes human HSPC self-renewal^11^. UM171, a pyrimidoindole derivative, stimulates ex vivo expansion of human HSPCs and attenuates cell differentiation^12^. Oct4-activating compound 1 (OAC1) increases numbers of human HSPCs by activating the Oct4-HOXB4 axis^13^. PGE2, a lipid signaling molecule, promotes amplification of HSPC^14^. SW033291, a small-molecule inhibitor, accelerates haematopoietic recovery in mice receiving a bone marrow transplant^15^. However, combinations of these molecules are untested.

Haematopoietic stem and progenitor cells are heterogeneous^16^. Prior analyses based on cell surface antigen staining are biased by limited choices of surface markers. Recently, single-cell transcriptome analyses were used to dissect cellular heterogeneity and construct lineage hierarchy in the haematopoietic system^17,18^. The behavior of human CD34-positive cells in the culture system has not been characterized at single-cell resolution.

In this study, we found that human CD34-positive cells can be maintained in vitro by a combination of CHIR-99021, Forskolin and OAC1 (CFO) without haematopoietic growth factors. Treatment increased numbers of phenotypic and functional human HSPCs. We characterized the underlying molecular events by single-cell RNA-seq analyses. We found clonal differences in the uncultured, CFO-cultured and HGF-cultured human CD34-positive cells. Our data suggests a new approach to maintain and possibly expand human CD34-positive cells for transplants and gene therapy.

## Results

### Chemical screening platform

**Fig. 1:**
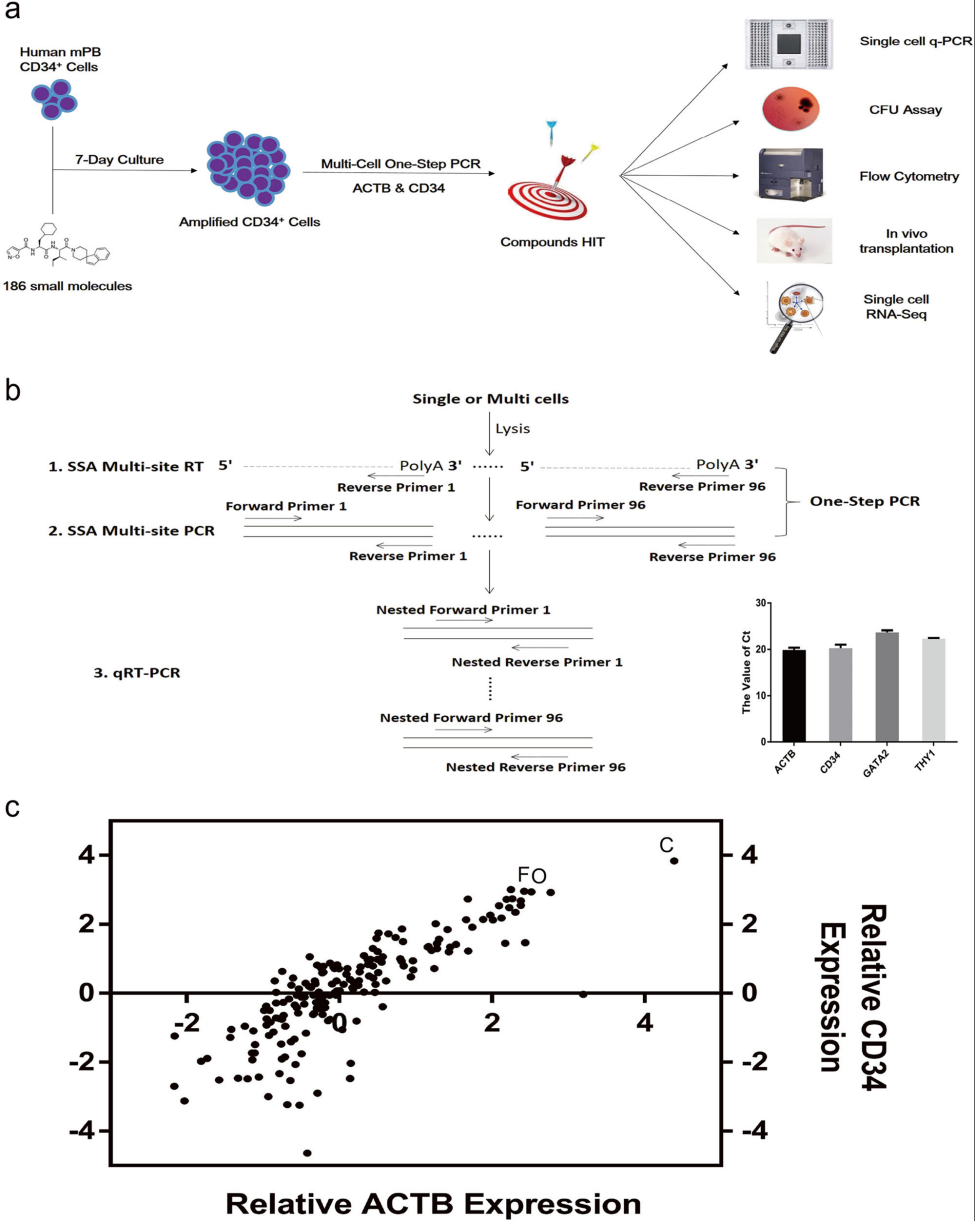
Chemical screening platform. **a** Framework of the experimental design. **b** Schematic diagram of multi-cell one-step PCR. Cells were collected into one tube containing enzymes and primers, frozen at –80 °C, and then underwent multi-site reverse transcription (RT) and sequence-specific amplification (SSA). The pre-amplified cDNA was ready for the subsequent qRT-PCR based gene quantification. Collection of 2,000 fresh human CD34-positive cells and detection of *ACTB*, *CD34*, *GATA2* and *THY1* transcript levels in HSPCs (bottom right corner). **c** A dot plot showing the result of primary chemical screening. Using the chemical screening platform, 2,000 human CD34-positive cells exposed to 186 individual small molecules were assayed for relative transcript expression of *ACTB* and *CD34*. IMDM supplemented with serum substitute served as control. C, CHIR-99021; F, Forskolin; O, OAC1

We designed a chemical screening platform to identify low molecular weight chemicals that support maintenance of functional human CD34-positive cells (Fig. 1a). First, we developed a multi-cell one-step PCR platform enabling efficient screening of chemical function on human HSPC maintenance. Cells were collected and sequence-specific amplification was performed on the common PCR instrumentation in 8−well PCR strips^19^. After the multi-site one-step reverse transcription (RT) and PCR, pre-amplified cDNA was used to quantify expression level of specific genes by qRT-PCR (Fig. 1b). We collected 2,000 fresh human CD34-positive cells and detected gene transcript levels using our multi-cell one-step PCR platform. Results show the value of Ct: *ACTB* (19.88 ± 0.51), *CD34* (20.30 ± 0.75), *GATA2* (23.68 ± 0.44) and *THY1* (22.35 ± 0.15) (Bottom right corner in Fig. 1b).

Using the platform, we screened 186 small chemicals for their ability to support human HSPC maintenance (Supplementary Table S1). We used Iscove Modified Dulbecco Medium (IMDM) and the serum substitute but excluded cytokines and HGFs. We found that human CD34-positive cells cultured with CHIR-99021 (C), a GSK-3 inhibitor, promoted an up to 3.84-fold increase in expression of human HSPC marker gene *CD34* (95% confidence interval [CI] 2.06, 5.61; *P* < 0.001) compared with controls. Cells cultured with Forskolin (F), an adenylyl cyclase activator (0.50, 5.40; *P* < 0.05) or with OAC1 (O), an induced pluripotent stem cell (iPSC) regulator (0.62, 5.26; *P* < 0.05) also enhanced levels of *CD34* transcripts compared with controls (Fig. 1c and Supplementary Table S1).

### CFO increases phenotypic and functional human HSPCs

**Fig. 2:**
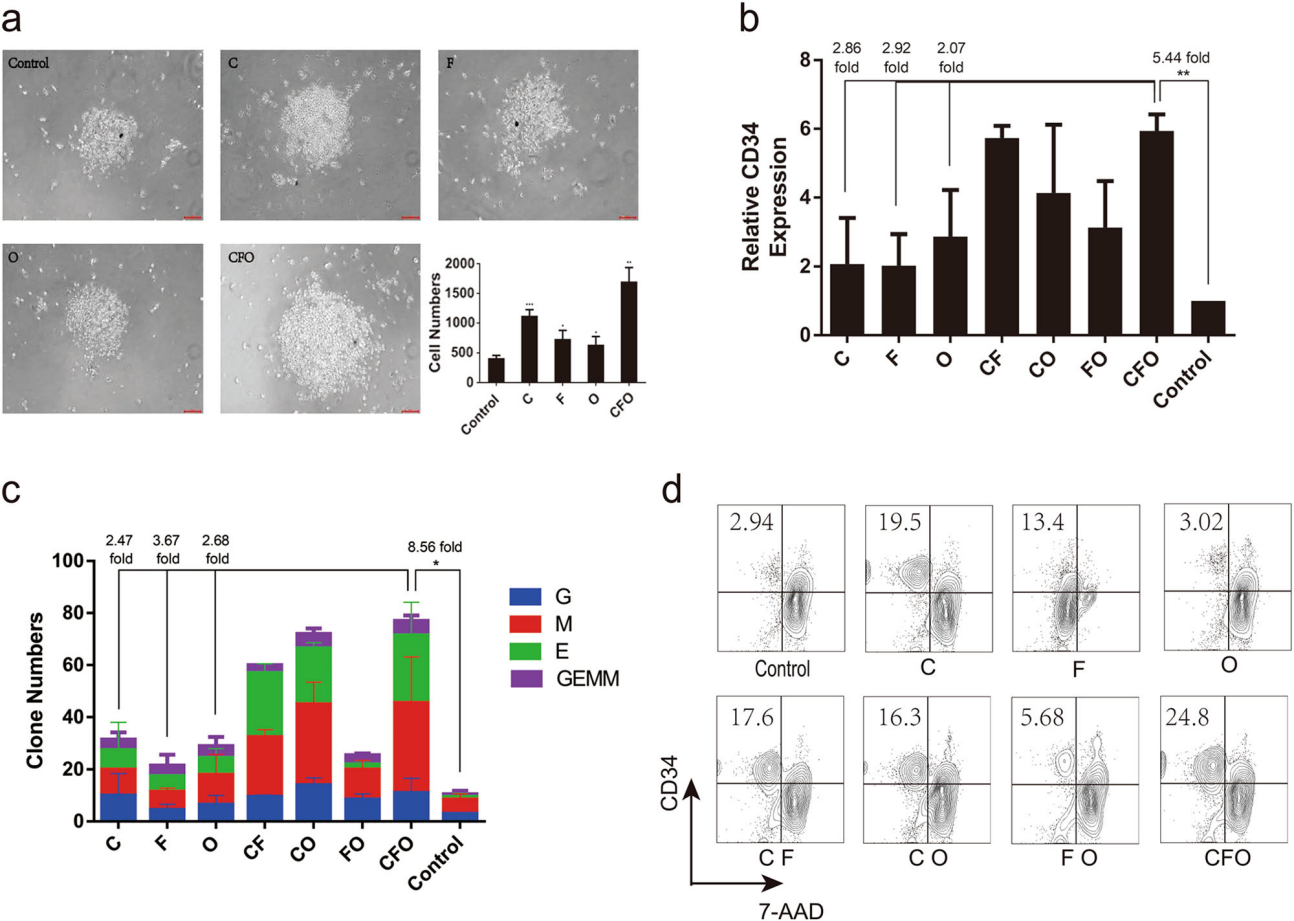
CFO increases phenotypic and functional human HSPCs. **a** Cell morphology and numbers. Numbers of human CD34-positive cells exposed to C, F, O and CFO were calculated (scale bar, 50 μm). *P < 0.05, **P < 0.01, ***P < 0.001. Data represents the mean ± SD. **b** Relative expression of *CD34*. Human CD34-positive cells exposed to C, F, O and combination were assayed for relative expression of *CD34*. **P < 0.01. Data represents the mean ± SD. **c** Clone numbers. Human CD34-positive cells exposed to C, F, O and combination were assayed for colony-forming units. G, granulocyte; E, erythroid; M, myeloid; GEMM, granulocyte, erythroid, monocyte and megakaryocyte colonies. **P < 0.01. Data represents the mean ± SD. **d** Phenotype of HSPC. Human CD34-positive cells exposed to C, F, O and combination were assayed for surface protein expression of CD3*4*. 7-AAD was assayed for dead cells. C, CHIR-99021; F, Forskolin; O, OAC1

We next designed experiments comparing effects of CFO on numbers of phenotypic and functional human HSPCs. We found that numbers increased by 4.09-fold (2.82, 5.36; *P* < 0.01) compared with controls. Control cultures contained mostly apoptotic cells after 7 days culture (Fig. 2a) and showed few CD34 transcripts. In contrast, transcript levels of *CD34* did not decrease when the culture medium contained CFO. Next, we tested various concentrations of these chemicals to determine their optimal concentrations, which were 10 μM (CHIR-99021), 20 μM (Forskolin), and 5 μM (OAC1). These concentrations were used in subsequent experiments (Supplementary Fig. S1a).

Cultures exposed to the combination of CFO contained significantly more granulocyte (CFU-G), myeloid (CFU-M), erythroid (CFU-E), and granulocyte, erythroid, monocyte, megakaryocyte (CFU-GEMM) progenitors after 14 days in vitro culture with 8.56-fold increase (7.09, 10.02; *P* < 0.05) compared with controls (Fig. 2c). Moreover, we observed that the proportion of *CD34*-positive cells was greatly enhanced by 8.1-fold (6.45, 9.75; *P* < 0.05) after cultures with the CFO compared with controls (Fig. 2d and Supplementary Fig. S1b).

### CFO maintains self-renewal of human HSPCs

**Fig. 3:**
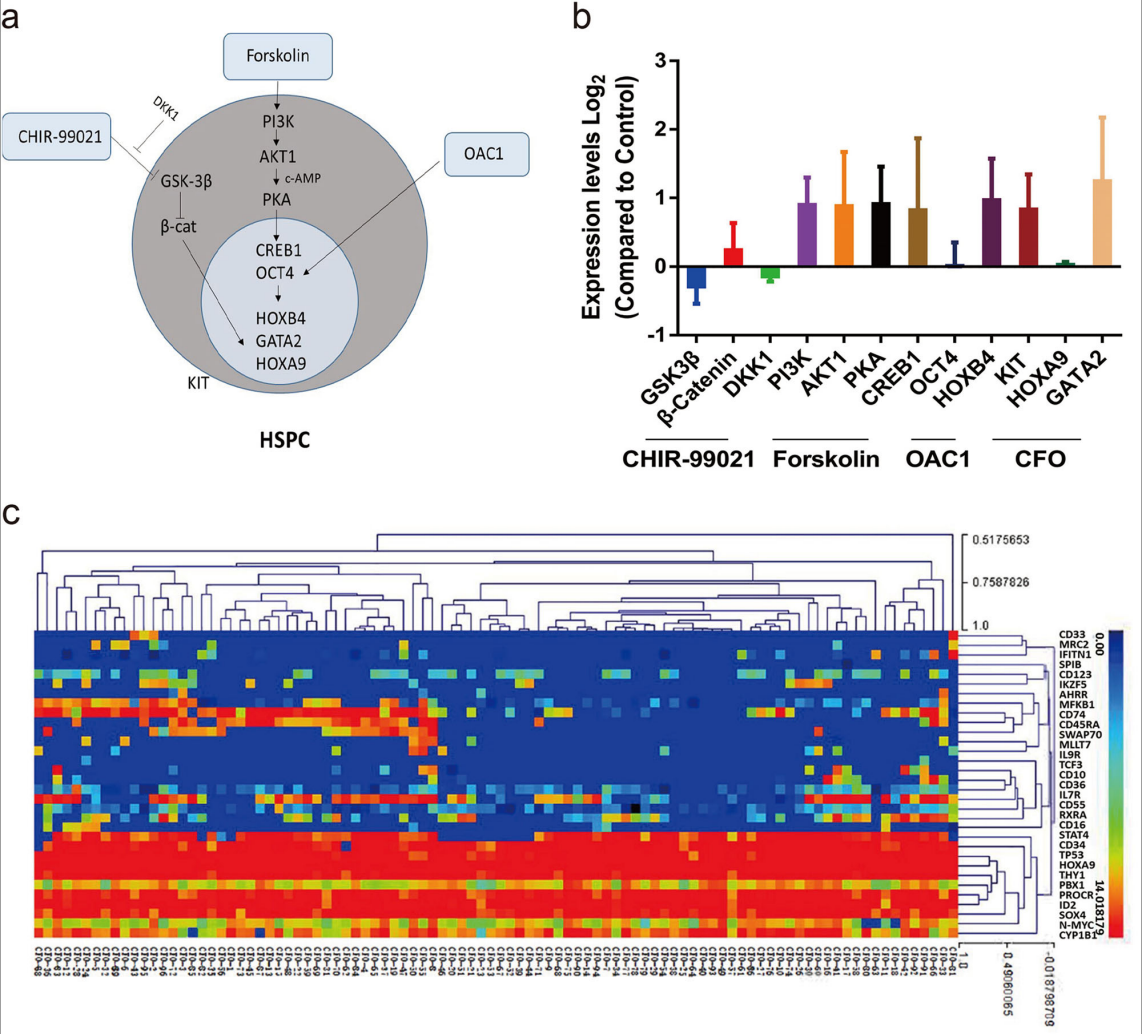
CFO maintains self-renewal of human HSPCs. **a** Schematic diagram of molecular regulators in HSPCs. Factors (CHIR-99021, Forskolin and OAC1) that have a demonstrated effect on human HSPCs are shown, including transcription factors and signaling pathways targeted for stem cell maintenance. **b** Changes in expression levels of CHIR-99021, Forskolin and OAC1 targets measured by qRT-PCR after a 7-day culture with CFO compared with control. Data represents the mean ± SD. **c** Gene expression profile of CFO. CFO group was randomly picked to perform single-cell qRT-PCR assay with selected genes from early human haematopoietic lineages. C, CHIR-99021; F, Forskolin; O, OAC1

It is well known that CHIR-99021 is a GSK-3 inhibitor, Forskolin is an adenylyl cyclase activator and OAC1 is an iPSC regulator^13,20^. After RNA isolation and qRT-PCR assay, we found that exposure of HSPCs to CFO activated the expression of *β-catenin*, *PI3K, AKT1, PKA, CREB1, OCT4, HOXB4, KIT, HOXA9 and GATA2* while suppressing the *GSK-3β* and *DKK1* levels, suggesting that CFO maintains human HSPC self-renewal by activating Wnt/β-catenin pathway, AKT-cAMP pathways and OCT4-HOXB4 axis (Fig. 3a, b).

To further evaluate effects of cultures with CFO, we randomly picked control and CFO cultured cells for single-cell qRT-PCR assay using 96 genes selected from early human haematopoietic lineages (Supplementary Table S2)^21^. In the clustering heatmap with control and CFO cultures, human HSPCs and differentiated cells were distinct (Fig. 3c and Supplementary Fig. S1d). Human CD34-positive cells cultured with CFO showed much higher transcript levels of human haematopoietic markers such as *CD34*, *SOX4*, *TAL1*, *N-MYC*, *HOXA9* and *THY1*. Control cells expressed higher level of genes associated with myeloid (*CD33*, *CD45RA*), erythroid (*RXRA*, *MLLT7*) and lymphoid (*IL7R*, *CD10*) differentiation (Supplementary Fig. S1c).

**Fig. 4:**
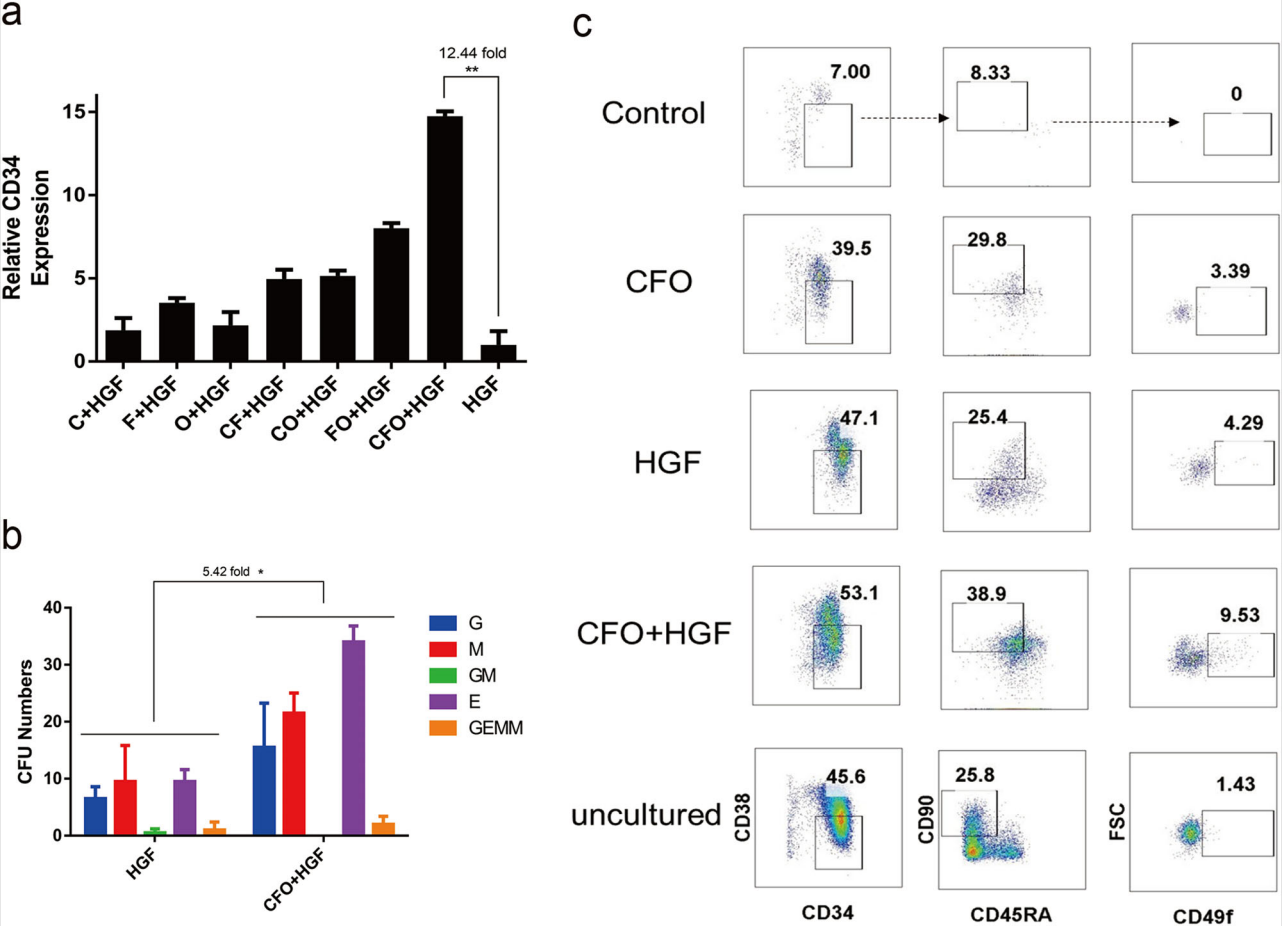
Combination of CFO and HGF increases functional human HSPCs. **a** Relative expression of *CD34*. Human CD34-positive cells exposed to C, F, O, CF, CO, FO and CFO on the basic medium with haematopoietic growth factor (HGF) were assayed for relative expression of *CD34*. *P < 0.05. Data represents the mean ± SD. **b** Clone numbers. Human CD34-positive cells exposed to CFO, HGF and CFO + HGF were assayed for colony-forming units. G, granulocyte; E, erythroid; M, myeloid; GM, granulocyte/monocyte; GEMM, granulocyte, erythroid, monocyte and megakaryocyte colonies. *P < 0.05. Data represents the mean ± SD. **c** Phenotype of HSC. Uncultured human CD34-positive cells and these cells exposed to CFO, HGF and CFO + HGF were assayed for CD34, CD38, CD45RA, CD90 and CD49f by flow cytometry. C, CHIR-99021; F, Forskolin; O, OAC1; HGF, stem cell factor (SCF) and thrombopoietin (TPO)

Next we added 2 HGFs, stem cell factor (SCF) and thrombopoietin (TPO) to culture with CFO for 7 days^1^. We observed a 3.57-fold (1.79, 5.35; *P* < 0.05) increase in HSPC numbers and > 12.44-fold (9.64, 15.24; *P* < 0.01) up-regulation of *CD34* transcript levels compared with cells exposed to these growth factors only (Fig. 4a and Supplementary Fig. S2a). Cells cultured with CFO + HGFs had a 5.42-fold (1.72, 10.12; *P* < 0.05) increase in CFU-Cs compared with HGFs only (Fig. 4b), but there was no difference in proportion of *CD34-*positive cells (Supplementary Fig. S2b).

Next, we studied effects of CFO on more primitive hematopoietic stem cells (CD34^+^CD38^-^CD45RA^−^CD90^+^CD49f^+^)^19^. We found that there were 1.43% CD34^+^CD38^−^CD45RA^−^CD90^+^CD49f^+^ cells in uncultured mobilized peripheral blood cells and 3.39% these cells with the CFO treatment. Besides, a 2.47-fold (1.40, 4.49; *P* < 0.05) increase in proportion of these cells in the CFO + HGFs treated group compared with HGFs only (Fig. 4c and Supplementary Fig. S2c).

### Effect of CFO on engraftment in immune-deficient mice

**Fig. 5:**
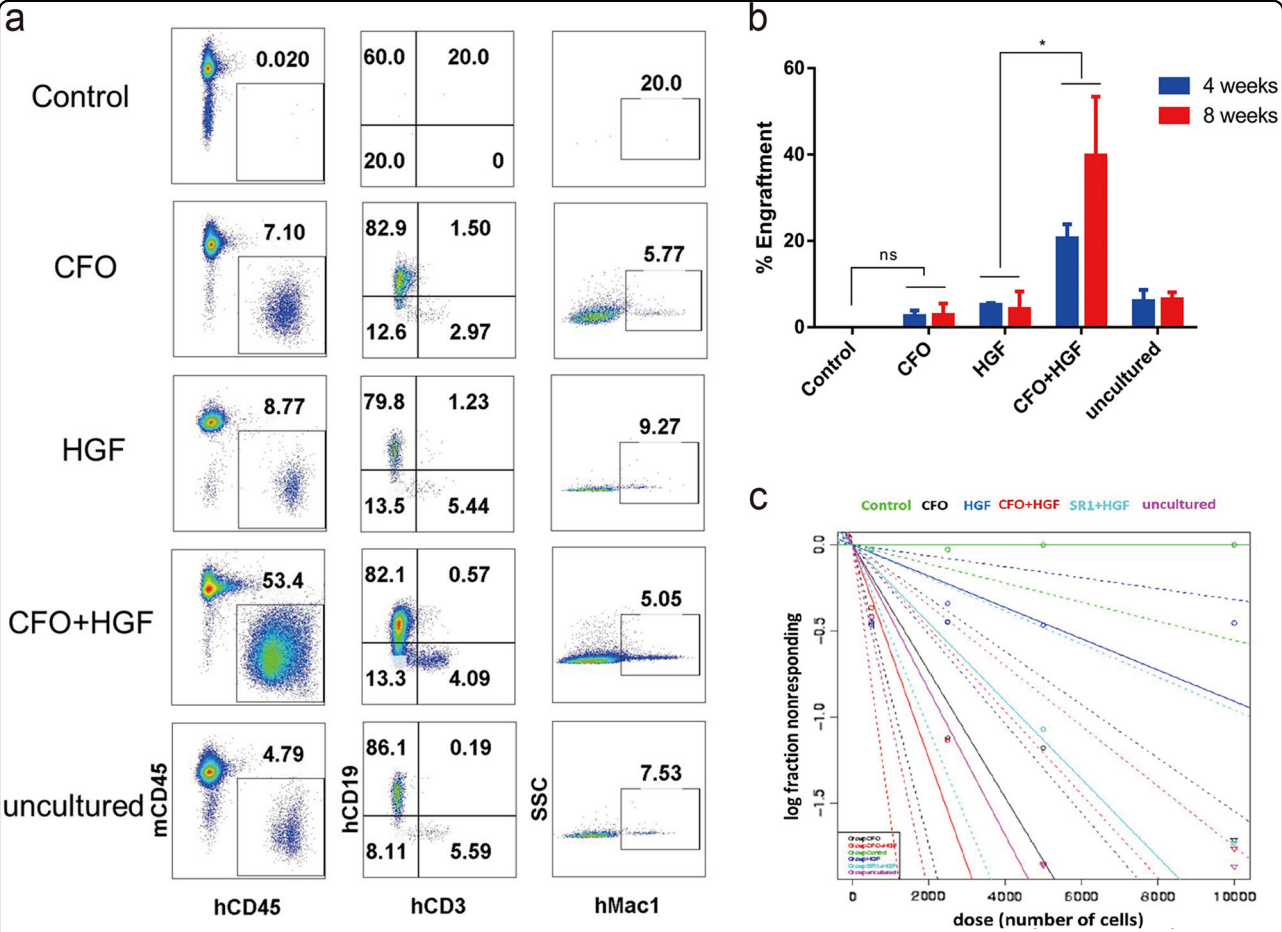
Effect of CFO on engraftment in immune-deficient mice. **a** Reconstruction of HSPCs in B-NSG mice. Flow cytometry plots show percentage of reconstituted human CD45^+^ leukocytes, CD3^+^ T cells, CD19^+^ B cells and Mac1^+^ myeloid cells in B-NSG mice with the injection of control cells, human CD34-positive cells exposed to CFO, HGF, CFO + HGF and uncultured cells. **b** Reconstruction of HSPCs in B-NSG mice after 4- and 8-week post-transplantation. *P < 0.05. Data represents the mean ± SD. ns, non-significant. **c** The frequency of human SRCs in uncultured HSPCs and the progeny of the equivalent number of cells treated with control, CFO, HGF, CFO + HGF or SR1. Poisson statistical analysis of data from Supplementary Table S3. Shapes represent the percentage of negative mice for each dose of cells. Solid lines indicate the best-fit linear model for each data set. Dotted lines represent 95% confidence intervals. C, CHIR-99021; F, Forskolin; O, OAC1; HGF, stem cell factor (SCF) and thrombopoietin (TPO)

To evaluate the functionality of CFO-treated human CD34-positive cells, sub-lethally irradiated NOD-Prkdc^scid^IL2rg^tm1^/Bcgen (B-NSG) immune-deficient mice were transplanted with 50,000 human CD34-positive cells cultured for 7 days with CFO, HGFs or both. As early as 4 weeks posttransplant we observed increased engraftment of human *CD45*-positive cells in the 3 settings compared with controls. By 8 weeks, engraftment of CFO-treated cells was still higher compared with controls, and cells cultured with CFO and HGFs was nearly 5-fold higher compared with HGF only cultures (*P* < 0.05, Fig. 5a, b). Moreover, cells cultured in CFO, HGFs or both showed multi-lineage reconstitution of human myeloid, T- and B-cells in the bone marrow (Fig. 5a and Supplementary Fig. S2d).

To further assess the degree of HSPC expansion by chemical cocktail treatment, we did a limiting dilution assay to compare the frequency of SCID Repopulating Cells (SRCs) in Day 0 uncultured HSPCs, in the progeny of an equivalent number of cells in the presence of control, CFO, HGF, CFO + HGF or SR1 + HGF after 7 days of culture. Poisson distribution analysis revealed an SRC frequency of 1/2,374 in Day 0 uncultured HSPCs, 1/2,726 in CFO cultures, 1/11,004 in HGF cultures, 1/1,615 in CFO + HGF cultures and 1/4,412 in SR1 + HGF cultures (Fig. 5c). We calculated the presence of 412 SRCs in 1 × 10^6^ Day 0 uncultured HSPCs, 367 SRCs, 91 SRCs, 619 SRCs and 227 SRCs in 1 × 10^6^ cells from CFO-, HGF-, CFO + HGF- and SR1 + HGF-treated cultures, respectively (Supplementary Table S3). Our data demonstrate that HSPCs cultured with CFO have a significant expansion of SRC numbers.

### Single-cell RNA-seq identify the mechanism of action

**Fig. 6:**
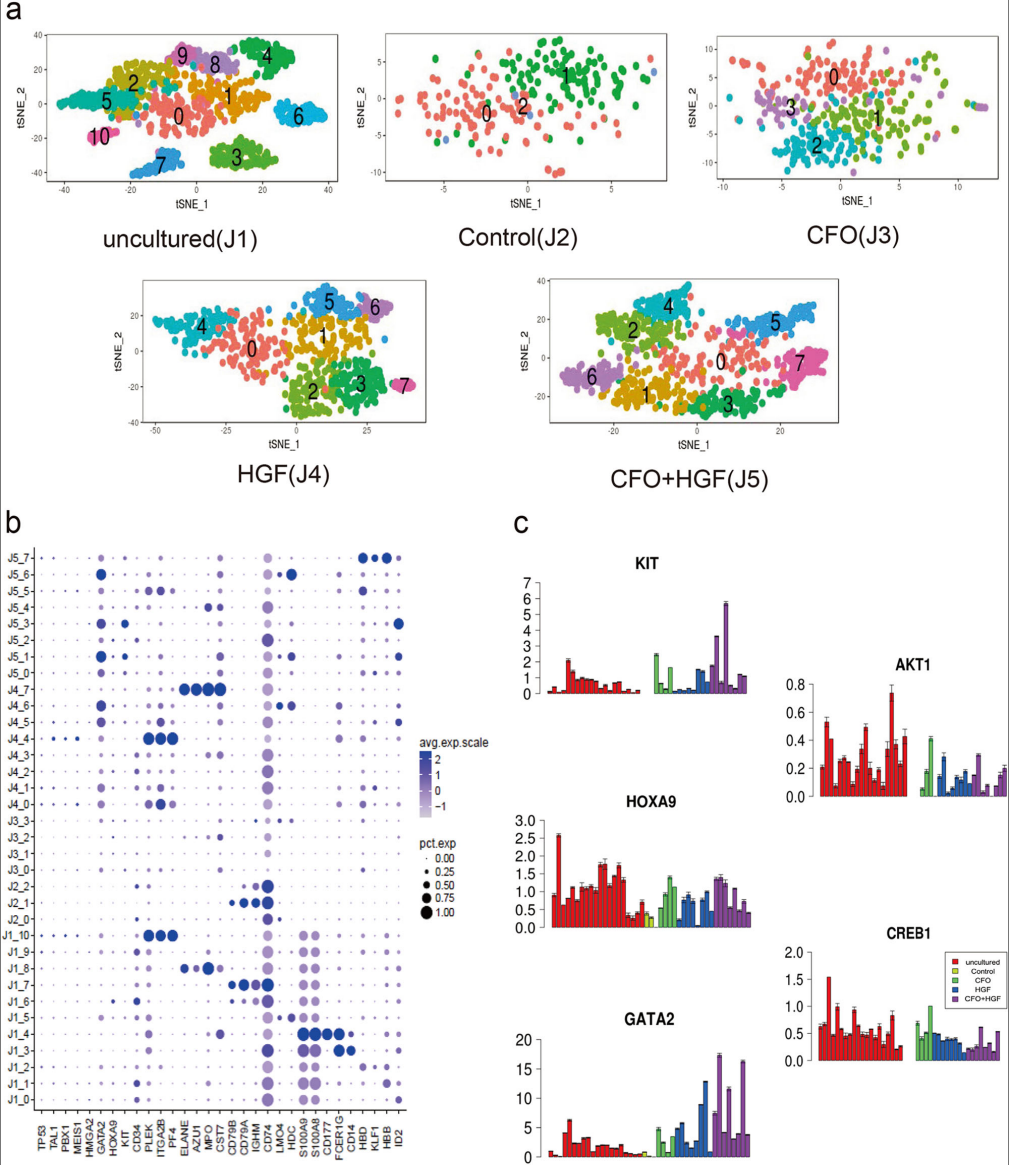
Single-cell RNA-seq identify the mechanism of action. **a** T-Distributed Stochastic Neighbor Embedding (t-SNE) analysis identified 11, 3, 4, 8 and 8 subpopulations in 5 samples: uncultured human CD34-positive cells (J1), control cells (J2), cells with the treatment of CFO (J3), HGF (J4) and CFO + HGF (J5). **b** Dot plot visualization of each subpopulation in the above 5 samples. The size of the dot encodes the percentage of cells within a subpopulation, while the color encodes the average expression level of ‘expressing’ cells. **c** Expression of selected genes across every subpopulation in the above 5 samples. 100 cells from each subpopulation were randomly sampled three times for gene expression analysis. C, CHIR-99021; F, Forskolin; O, OAC1; HGF, stem cell factor (SCF) and thrombopoietin (TPO)

We used our microwell single-cell RNA-seq platform^22–24^ to analyze fresh human CD34-positive cells, uncultured cells (J1), control-cultured cells (J2), CFO-cultured cells (J3), HGF-cultured cells (J4) and cells cultured with CFO + HGFs (J5). An average of 4,000 single cells were analyzed for each population. Samples were divided into 11, 3, 4, 8 and 8 subpopulations, respectively, using t-Distributed Stochastic Neighbor Embedding (t-SNE) analysis (Fig. 6a). Heatmap analyses revealed specific gene expression modules associated with each cluster (Supplementary Fig. S3a). Cluster-specific gene expression patterns for these populations are shown in Fig. 6b. For instance, a novel dendritic cell progenitor transcribing *ID2* was found in Cluster 0 (C0) of uncultured cells (J1_0), C5 of HGF-cultured cells (J4_5) and C3 of cells cultured with CFO + HGF (J5_3). Erythroid progenitors exhibited high levels of *HBB* and *HBD* transcripts in J1_1, J1_2, J5_5 and J5_7. B cell progenitors correspond to J1_7, J2_1 and J2_2, with specific markers of *CD79A, CD79B* and *IGHM*. J1_8 and J4_7 showed higher level of *MPO, AZU1* and *ELANE* transcripts, consistent with granulocyte progenitors. J1_10 and J4_4 displayed megakaryocyte-related transcripts with high expression of *ITGA2B* (*CD41*), *PLEK* and *PF4*. More data are displayed in Supplementary Table S4.

To explore regulatory models, we randomly sampled 100 cells twice from each population and used aggregated data for network interpretation^25^. Cells cultured with CFO with and without HGF had higher transcript levels of *KIT* (a surface marker) and *GATA2* and *HOXA9* (transcription regulators) compared with controls. We also found that CD34-positive cells cultured with CFO had up-regulated self-renewal via the AKT-cAMP signaling pathway which activates *AKT1* and *CREB1* (Fig. 6c).

## Discussion

Combined CHIR-99021, Forskolin and OAC1 (CFO) maintains and perhaps increases numbers of human HSPCs in vitro. CFO cultures of CD34-positive cells showed increased expression of HSPC markers and increased haematopoietic repopulating ability in immune-deficient mice. Using single-cell RNA-seq analysis, we found that CFO supports HSPC maintenance and possibly self-renewal by activating transcription factor *HOXA9* and *GATA2*, as well as the AKT-cAMP signaling pathway.

In primary screening, 186 small molecules were selected to maintain HSPC in vitro. Among them, 35 compounds were TGF-β/Smad inhibitor. From homeostasis of the immune system to quiescence and self-renewal of HSCs, TGF-β signaling controls a wide spectrum of biological processes in the hematopoietic system^26^. Twenty-nine low-molecule-weight compounds were related to JAK/STAT signaling, which plays an important role in the hematopoietic cell lineages^27^. Sixteen small molecules were related to the Wnt/β-catenin pathway. Genetic and chemical manipulation of Wnt signaling has been shown to affect HSC expansion^28^. Ten compounds correspond to ROCK inhibitor, 8 were Hedgehog/Smoothened receptor antagonist and the rest were also related to stem cell development. In our study, CHIR-99021, a GSK-3 inhibitor, Forskolin, an adenylyl cyclase activator and OAC1, an iPSC regulator, were hit to maintain HSPC in vitro.

We used a multi-cell one-step PCR platform for the primary chemical screen, a method adopted from the single-cell qRT-PCR system^29^. Using this technique we detected *ACTB*, *CD34*, *GATA2*, and *THY1 (CD90)* in human CD34-positive cells, suggesting efficacy of this strategy for high-throughput gene expression analysis. Besides *CD34* expression, we also measured the expression of *GATA2* and *CD90* after 7-day culture in vitro. We found that transcript expression of *GATA2* and *CD90* was similar to *CD34*, but transcript expression of *CD34* was more stable and dominant. Hence, we choose the transcript expression of *CD34* to assess efficiency of chemicals. Moreover, our data suggests that CFO is more efficient than 1 or 2 chemicals in maintaining human HSPC in vitro. Combining CFO with HGFs also increased efficacy in almost all assays. In transplantation assay, we found that the CFO + HGF-treated groups resulted in detectable engraftment of human CD45^+^ cells in secondary mouse recipients (Supplementary Fig. S2c).

Single-cell RNA-seq is a powerful tool for studying complex biological systems such as human HSPCs cultured in vitro. By focusing on effects of each chemical, we found that control CD34-positive cells were prone to B-cell differentiation. This was not seen in CD34-positive cells cultured with CFO. CFO-cultured CD34-positive cells expressed the same cell surface marker modules and transcription factors as fresh CD34-positive cells (Fig. 6b and Supplementary Fig. S4)^21^. Furthermore, we detected genes enriched in cell proliferation and anti-apoptotic processes in CD34-positive cells cultured with CFO (Supplementary Fig. S3b).

Previous studies have discovered different regulation mechanisms in expansion of human HSPCs in vitro. SR1 treatment resulted in down-regulation of aryl hydrocarbon receptor (AhR) target genes such as *CYP1B1*, *CYP1A1*, and *AhRR*^11^. Besides, RNA-binding protein Musashi-2 (MSI2) directly attenuates AHR signaling through post-transcriptional down-regulation to enhance the regenerative potential of human HSPCs ex vivo^30^. Unlike SR1, HSPCs with the treatment of UM171 were accompanied by a marked suppression of transcripts associated with erythroid and megakaryocytic differentiation^12^. Moreover, through OCT4-mediated up-regulation of *HOXB4*, OAC1 could enhance ex vivo expansion of human HSPCs^13^. In our study, single-cell RNA-seq revealed that HSPC cultured with CFO significantly activated the expression of *KIT, HOXA9, GATA2, AKT1*, and *CREB1* (Fig. 6c). On the one hand, CHIR-99021 is a GSK3 inhibitor, Forskolin is an adenylyl cyclase activator and OAC1 is an iPSC regulator. CFO treatment activated the Wnt/β-catenin and AKT-cAMP signaling pathway in maintenance of HSPCs in vitro. On the other hand, CHIR99021, Forskolin and OAC1 are three known compounds in reprogramming progress^8^. We hypothesized that CFO could induce differentiated cells into reprogrammed HSPCs with high level of *KIT*, *HOXA9*, and *GATA2*.

In conclusion, we found that human HSPCs are maintained in vitro in cultures with CFO. Using chemicals to maintain or increase HSPCs offers a new approach to solve problems in haematopoietic cell transplants and gene therapy.

## Materials and methods

### Human CD34-positive blood cells

Recombinant human granulocyte colony-stimulating factor (G-CSF) mobilized blood samples were collected from healthy donors at The First Affiliated Hospital of Zhejiang University School of Medicine (Zhejiang, China). Participants gave written informed consent. Procedures are approved by the Ethical Committee on Medical Research at School of Medicine. Human CD34-positive cells were isolated using EasySep^TM^ (STEMCELL Technologies, Vancouver, Canada) according to the manufacturer’s protocol.

### Chemical screening and human HSPC cultures

In total, 186 chemicals were screened including 150 chemicals from the Stem Cell Library (Target Mol, Shanghai, China) and 36 chemicals from our previous studies^7^ (Selleck Chemicals, Shanghai, China). Human CD34-positive cells were cultured in IMDM (STEMCELL Technologies) supplemented with serum substitute (STEMCELL Technologies). Human stem cell factor (SCF, PeproTech, Rocky Hill, NJ, US; 100 ng/mL) and thrombopoietin (TPO, PeproTech; 50 ng/mL) were added to the medium. Human CD34-positive cells were re-suspended in culture medium (2,000 cells/40 μL) and distributed into 96-well plates.

### Multi-cell one-step PCR assay

Gene expression of human CD34-positive cells was determined after 7 days of culture using multi-cell one-step PCR. Amplified human CD34-positive cells were transferred into 8-well PCR strips loaded with One-Step PCR Master Mix in each well (Vazyme, Nanjing, China) and strips frozen at –80 °C for 5 min. Plates were placed in the PCR machine after brief centrifugation. Cell lyses and sequence-specific reverse transcription were performed at 50 °C for 60 min. Reverse transcriptase inactivation and Taq polymerase activation were achieved by heating to 95 °C for 3 min. Subsequently, cDNA was subjected to 10 cycles of sequence-specific amplification by denaturing at 95 °C for 15 s, annealing and elongation at 60 °C for 15 min. Amplified cDNA was used for qRT-PCR. Detection of gene expression from the amplified cDNA was performed using LightCycler^®^ 480 (Roche, Basel, Switzerland). To detect optimal concentrations of CHIR-99021, Forskolin and OAC1, double (20 μM) or half (5 μM) concentration of each small-molecule combination on the basic of CFO (10 μM) was assayed for *CD34* transcript levels.

### Colony-forming unit assay

Frequencies of colony-forming cells were estimated by plating cultured human CD34-positive cells into methylcellulose-based medium with recombinant cytokines (STEMCELL Technologies). Three independent experiments were performed for each population. After 14 days of culture, multi-lineage colonies were enumerated under an inverted microscope (Nikon, Tokyo, Japan).

### Flow cytometry

Cultured cells were stained in PBS supplemented with 2% fetal bovine serum (FBS) at 4 °C for 30 min with the following human antibodies: CD34 PE (Biolegend, clone 581, Santiago, CA, US), CD34 FITC (BD Biosciences, clone 581, Franklin Lake, NJ, US), CD38 PE-Cy7 (BD Biosciences, clone HIT2), CD90 APC (BD Biosciences, clone 5E10), CD45 PE (Biolegend, clone HI30), CD11b/Mac1 APC (BD Biosciences, clone ICRF44), CD3 FITC (BD Biosciences, clone HIT3a), CD19 BV201 (Biolegend, clone SJ25C1), anti-mouse CD45 PerCP-Cy5.5 (Biolegend, clone 30-F11) and 7-amino-actinomycin D (7-AAD) (Biolegend). 7-AAD was used to exclude dead cells. Stained cells were washed once with PBS supplemented with 2% FBS and analyzed using the BD LSRFortessa (BD Biosciences). Proportion of positive/negative cells with the same mean fluorescence intensity (MFI) was represented.

### RNA isolation and qRT-PCR analysis

RNA was extracted using EasyPure RNA Kit (TransGen, Beijing, China) according to the manufacturer’s instructions. Purified RNA was subjected to cDNA synthesis using TransScript All-in-One First-Strand cDNA Synthesis SuperMix for qPCR (TransGen) according to the manufacturer’s instructions. The cDNA served as a template for the amplification of *GSK-3β, β-catenin, DKK1, PI3K, AKT1, PKA, CREB1, OCT4, HOXB4, KIT, HOXA9* and *GATA2* by real-time PCR, using a 384-well plate in a total volume of 10 μL which contained 1.4 μL of cDNA, 0.3 μL of primer at 10 μM, 3.3 µL H_2_O and 5 μL of SYBR Green Master Mix (Vazyme). Reactions were amplified on LightCycler^®^ 480 (Roche) using standard parameters.

### Single-cell gene expression analysis by micro-fluidic qRT-PCR

Ninety-six individual primer sets were pooled to a final concentration of 0.1 μM for each primer as described^29^. After 7 days of culture, 96 single cells were randomly picked from cultures incubated with control or the combination of CHIR-99021, Forskolin and OAC1 conditioned medium and sorted into 8-well PCR strips loaded with 5 μL RT-PCR Master Mix (Vazyme) in each well. Sorted strips were immediately frozen at –80 °C and immediately placed into the PCR machine after brief centrifugation. The PCR progress was identical to the multi-cell one-step PCR but with 20 cycles of sequence-specific amplification. After pre-amplification, PCR strips were stored at –80 °C to avoid evaporation. Pre-amplified products were diluted by 5-fold and analyzed with EvaGreen 2 × qPCR MasterMix (Applied Biological Materials, Vancouver, Canada), 20 × DNA Binding Dye (Fluidigm, San Francisco, CA, US) and individual qPCR primers using 96.96 Dynamic Arrays on a BioMark System (Fluidigm). Threshold crossing (Ct) values were calculated using the BioMark Real-Time PCR Analysis software (Fluidigm).

### Engraftment of human HSPCs in B-NSG mice

B-NSG (NOD-Prkdc^scid^IL2rgtm1/Bcgen, Biocytogen, Beijing, China) mice were maintained in the Laboratory Animal Center of Zhejiang University. Animal experiments were conducted under protocols approved by the Ethical Committee on Laboratory Animal Center of Zhejiang University. 50,000 uncultured human CD34-positive cells and their progenies from 7-day cultures with the combination of CHIR-99021, Forskolin and OAC1 only, with HGF only or both were injected into tibias of 6-8-week-old female B-NSG mice after exposing the mice to 2 Gy (Rad Source Technologies, Buford, GA, US). Engraftment efficiency was measured by flow cytometry analyses of bone marrow samples as described above. For long-term engraftment assay, bone marrow cells from the primary recipient mice were infused into secondary recipient mice. Bone marrow cells were stained and analyzed by flow cytometry as described above.

### Limiting dilution analysis

The frequency of human SRC in uncultured HSPCs and the progeny of an equivalent number of HSPCs that were ex vivo cultured in the presence of control, CFO, HGF, CFO + HGF or SR1 + HGF were analyzed by limiting dilution assay. Increasing doses of uncultured HSPCs (500, 2500, 5000, 10,000) or the progeny of an equivalent number of HSPCs were infused into B-NSG mice. These mice were sacrificed at 8 weeks after transplantation. The HSPC frequency was calculated and plotted using ELDA software (bioinf.wehi.edu.au/software/elda/).

### Single-cell RNA-seq

Single-cell RNA-seq experiments were performed using a home-made Microwell-seq platform as described^31^. Briefly, barcoded beads and single cells were blocked in an array of agarose micro-wells enabling efficient cell lysis and transcript capture. Template switch was performed using Smart-seq 2^32^. Briefly, 20 μL of RT mix was added to the collected beads. The RT mix contained 200 U SuperScript II reverse transcriptase, 1 × Superscript II first-strand buffer (Takara Bio, Shiga, Japan), 20 U RNase Inhibitor (Sangon, Shanghai, China), 1 M betaine (Sigma, San Francisco, CA, US), 6 mM MgCl_2_ (Ambion, America), 2.5 mM dithiothreitol, 1 mM dNTP and 1 μM TSO primer (Sangon). Amplified cDNAs were fragmented by a customized transposase that carries two identical insertion sequences (Vazyme). 3′ ends of transcripts were enriched in the library generation PCR and sequenced using Illumina Hiseq platforms according to the manufacturer’s protocol (Santiago, CA, USA).

### Statistical analysis

Results are expressed as mean values ± standard deviation (SD). *P*-value < 0.05 (two-tailed Student’s *t*-test) was considered significant. The pre-process of single-cell RNA-seq raw data was performed following drop-seq core computational tool^22^. Cell barcode and unique molecular identifier were extracted before reads alignment by STAR^33^. The clustering algorithm for the data was implemented and performed using Seurat R toolkit for single-cell genomics^34^. Principal components analysis (PCA) was performed and individual cells were clustered onto a single two-dimensional map using t-SNE analysis based on their PC scores. Clusters with specific markers were visualized on heatmaps. 100 cells from each cluster were randomly sampled three times for further genetic network analysis. Identification of enriched biological themes was achieved through Gene Ontology Consortium. RNA-seq data are available in GEO under accession number GSE107517.

## Electronic supplementary material

Supplemantary Figures

Supplementary information, Table S1

Supplementary information, Table S2

Supplementary information, Table S3

Supplementary information, Table S4

## Electronic supplementary material

**Supplementary Information** accompanies the paper at (10.1038/s41421-018-0059-5).
