# Supplementary material for: Maintenance of human haematopoietic stem and progenitor cells in vitro using a chemical cocktail
Source: Cell Discov. 2018 Oct 30;4:59. doi: 10.1038/s41421-018-0059-5 (PMC6206058; doi:10.1038/s41421-018-0059-5)
Supplement: Supplementary file 1 — Supplemantary Figures [file 41421_2018_59_MOESM1_ESM.pdf]

# Supplementary Figure 1

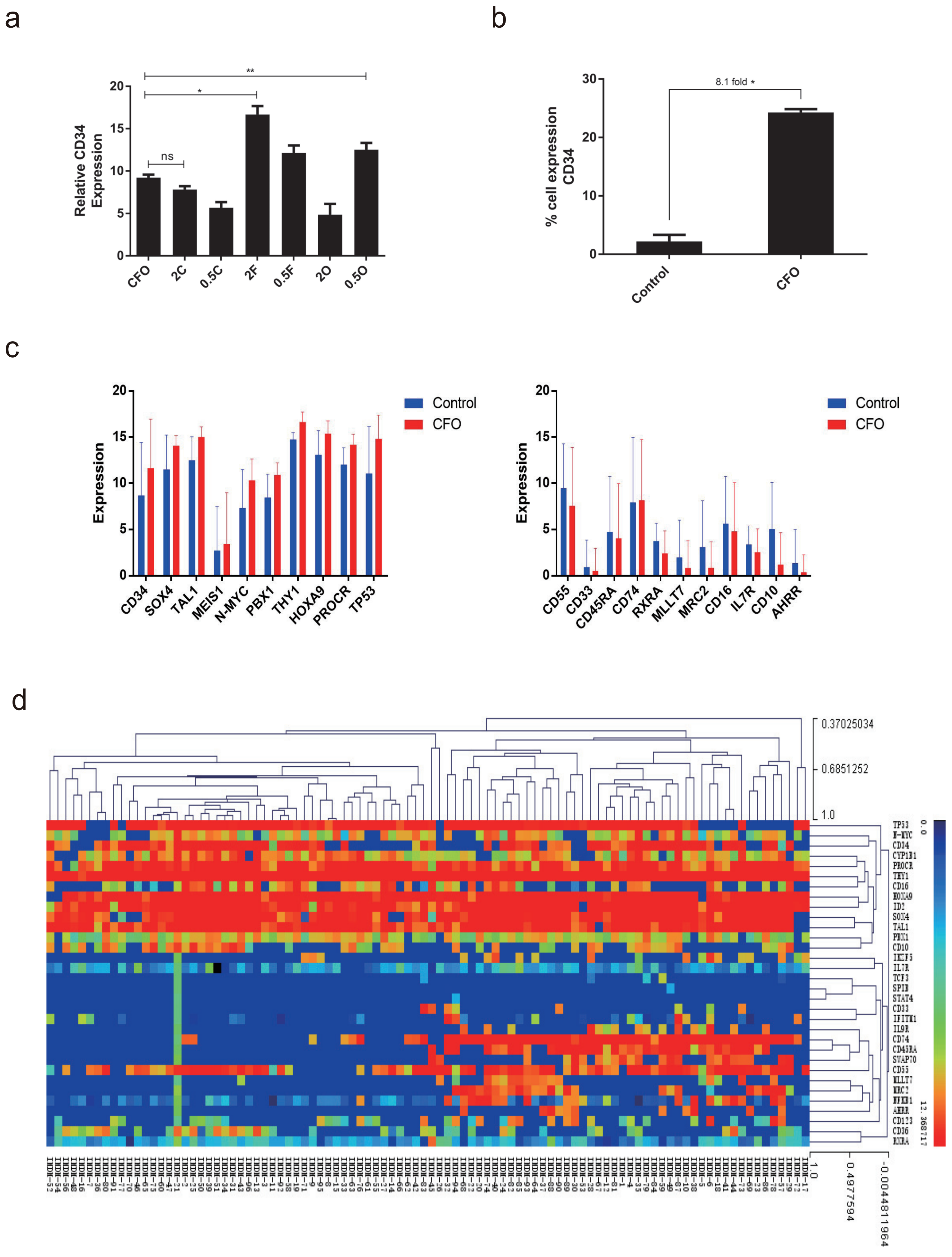

**Supplementary Figure S1** (Related to Figure 2 and Figure 3): **CFO maintains human HSPCs self-renewal.**

(a) Small molecule concentration adjustment. Experiments with double or half concentration of C, F and O determined the optimal combination for C (10 μM), F (20 μM) and O (5 μM). $**P < 0.01$, $*P < 0.05$. Data represents the mean ± s.d. ns, non-significant. (b) Quantification of the percentage of human CD34-positive cells showing *CD34* expression. Human CD34-positive cells exposed to CFO combination were assayed for surface protein expression of *CD34* by flow cytometry. $**P < 0.01$. Data represents the mean ± s.d. (c) Relative expression of related genes in control and CFO. HSPC related markers such as *CD34* and *HOXA9*, differentiated genes such as myeloid (*CD33*, *CD45RA*), erythroid (*RXRA*, *MLLT7*) and lymphoid (*IL7R*, *CD10*) were detected in control and CFO combination. Data represents the mean ± s.d. (d) Gene expression profile of control. Control were randomly picked to perform single cell qRT-PCR assay with selected genes from early human haematopoietic lineages. IMDM supplemented with serum substitute as control. C, CHIR-99021; F, Forskolin; O, OAC1.

# Supplementary Figure 2

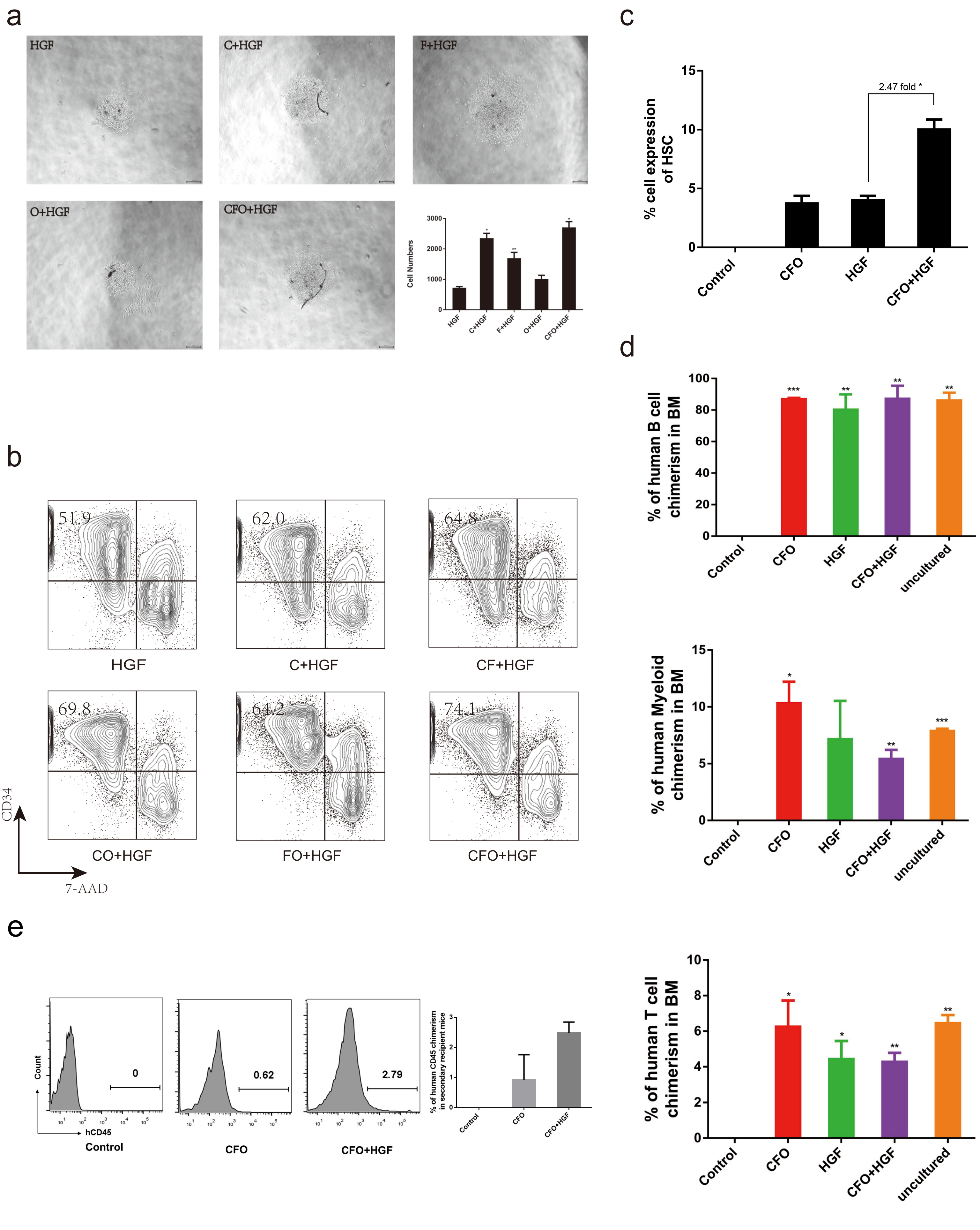

**Supplementary Figure S2** (Related to Figure 4 and Figure 5): **Combination of CFO and HGF increases functional human HSPCs.**

(a) Cell morphology and numbers. Numbers of human CD34-positive cells exposed to C, F, O and CFO on the basic medium with haematopoietic growth factors (HGF) were calculated (scale bar, 100 μm). ***P* < 0.01, **P* < 0.05. Data represents the mean ± s.d. (b) Phenotype of HSPCs. Human CD34-positive cells exposed to C, FO, CF, CO and CFO on the basic medium with HGF were assayed for surface protein expression of *CD34*. 7-AAD was assayed for dead cells. (c) Quantification of the percentage of human HSPCs showing *CD34* and *CD90* expression. Human CD34-positive cells exposed to CFO, HGF and CFO + HGF were assayed for surface protein expression of *CD34* and *CD90* by flow cytometry. **P* < 0.05. Data represents the mean ± s.d. (d) Chimerism of human myeloid cells, T cells and B cells in B-NSG mice with the injection of control cells, Human CD34-positive cells exposed to CFO, HGF, CFO + HGF and uncultured cells after 8-week post-transplantation. ****P* < 0.001, ***P* < 0.01, **P* < 0.05. Data represents the mean ± s.d. (e) Representative flow cytometric analysis of secondary engraftment in the bone marrow of B-NSG mice. Human engraftment was assessed as the percentage of human $CD45^+$ cells. Data represents the mean ± s.d. C, CHIR-99021; F, Forskolin; O, OAC1; HGF, stem cell factor (SCF) and thrombopoietin (TPO).

# Supplementary Figure 3

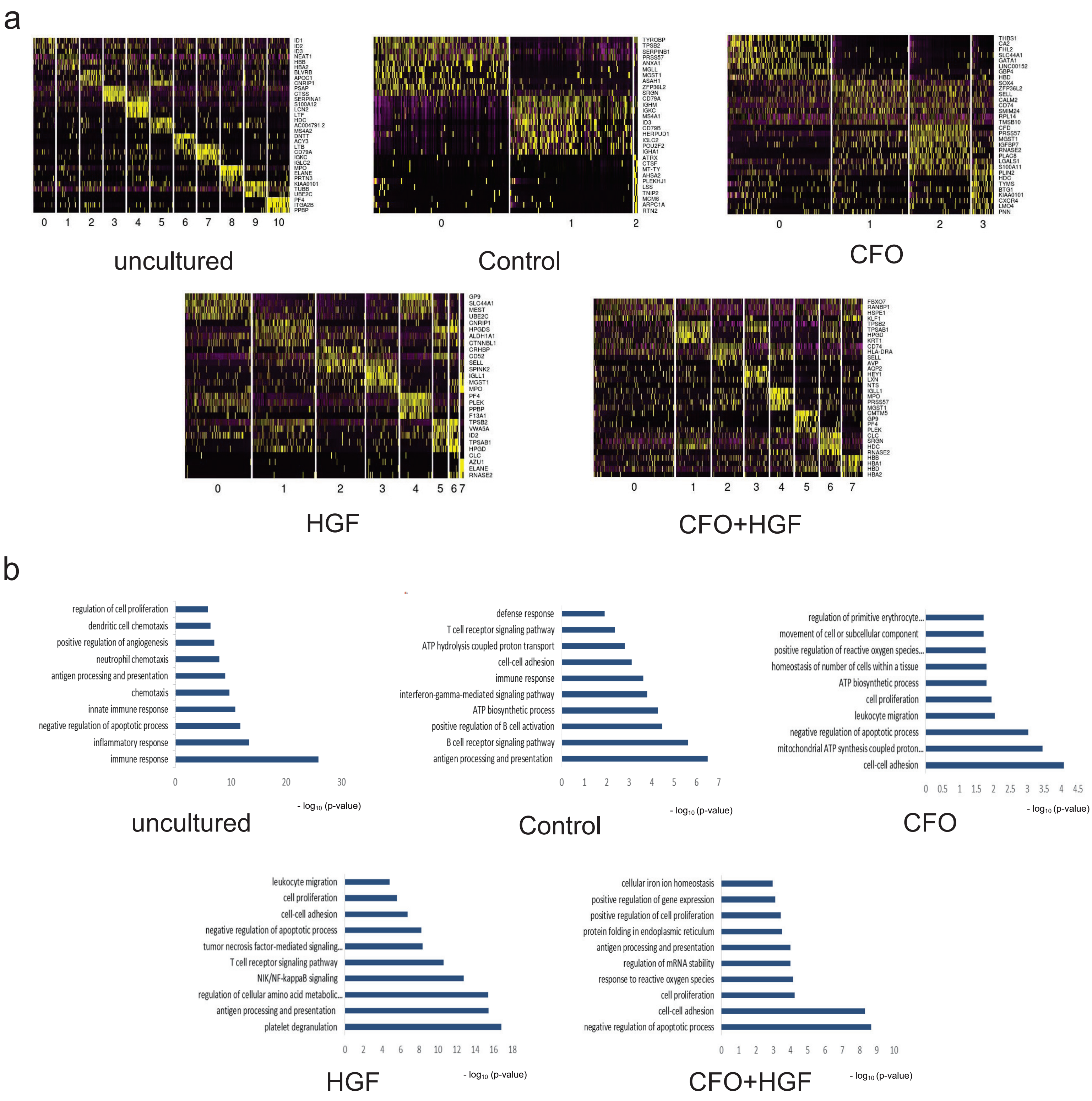

**Supplementary Figure S3** (Related to Figure 6): **Single cell RNA-seq identify the mechanism of action.**

(a) Heatmap of 5 samples: uncultured human CD34-positive cells, control cells, cells with the treatment of CFO, HGF and CFO + HGF. Heatmap reveals specific gene expression modules associated with each cluster. (b) Gene Ontology analysis. GO analysis revealed gene function and relationships in above 5 samples. C, CHIR-99021; F, Forskolin; O, OAC1; HGF, stem cell factor (SCF) and thrombopoietin (TPO).

# Supplementary Figure 4

a

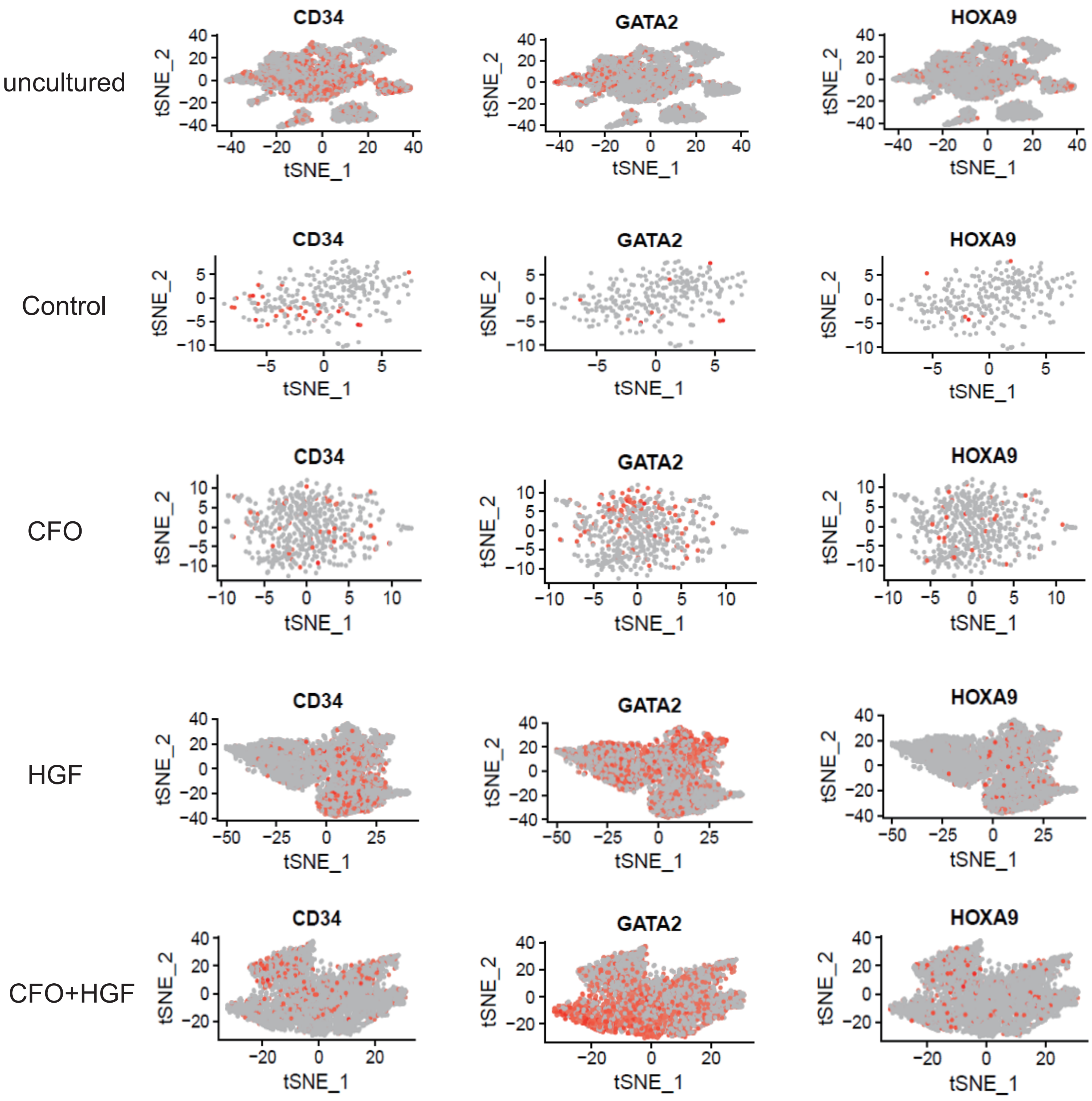

**Supplementary Figure S4** (Related to Figure 6): **Single cell RNA-seq identify the mechanism of action.**

(a) T-Distributed Stochastic Neighbor Embedding (t-SNE) analysis of *CD34*, *GATA2* and *HOXA9* in 5 samples: uncultured human CD34-positive cells, control cells, cells with the treatment of CFO, HGF and CFO + HGF. Red plots represent highly expressed cells. C, CHIR-99021; F, Forskolin; O, OAC1; HGF, stem cell factor (SCF) and thrombopoietin (TPO).
